# Effect of Demineralization on Fatigue-Based Shear Bond Strength Across Different Orthodontic Brackets: An In Vitro Study

**DOI:** 10.3390/jcm15062136

**Published:** 2026-03-11

**Authors:** Taylan Aydoğan, Orhan Cicek, Mehmet Yetmez

**Affiliations:** 1 Department of Orthodontics, Faculty of Dentistry, Zonguldak Bülent Ecevit University, Zonguldak 67600, Türkiye; taylan.aydogan@beun.edu.tr; 2 Department of Mechanical Engineering, Faculty of Engineering, Zonguldak Bülent Ecevit University, Zonguldak 67100, Türkiye; yetmez@beun.edu.tr

**Keywords:** orthodontic brackets, enamel demineralization, fatigue-based shear bond strength, cyclic loading, shear strokes to failure, enamel and dentin damage

## Abstract

**Background/Objectives**: Demineralization around orthodontic brackets may compromise enamel integrity and alter the mechanical stability of the bracket–adhesive–enamel interface, thereby influencing bond performance and clinical outcomes. This study aimed to evaluate the effect of enamel demineralization on the fatigue-based shear bond strength (SBS) of different orthodontic brackets. **Methods**: Seventy-five extracted maxillary premolars subjected to demineralization were allocated into five groups (*n* = 15 per group). Victory metal (Group 1), APC Clarity Advanced ceramic (Group 2), Clarity Self-ligating ceramic (Group 3), Gemini metal (Group 4), and Clarity Advanced ceramic (Group 5) brackets were bonded to the tooth surfaces using Transbond XT (3M Unitek, Monrovia, CA, USA). The mean demineralization values of the specimens were recorded before demineralization (T0) and after exposure to an artificial cariogenic environment (T1). Fatigue-based SBS was evaluated under cyclic loading (10 N, 0.5 Hz) at a crosshead speed of 300 mm/min using a closed-loop controlled, low-cycle fatigue testing machine and expressed as the number of shear strokes to failure. The level of statistical significance was set at *p* < 0.05. **Results:** No significant differences in demineralization were observed among the groups at T0 (*p* > 0.05); however, all groups showed significant increases at T1 (*p* < 0.05), with Group 1 demonstrating significantly lower demineralization than the other groups (*p* < 0.05). Fatigue-based SBS was higher in Groups 1, 3, and 5 than in Groups 2 and 4, as indicated by a greater number of shear strokes to failure (*p* < 0.05). In Groups 2 and 4, a statistically significant negative correlation was observed between changes in enamel demineralization and the number of shear strokes to failure (*p* < 0.05). No hard tissue damage was observed in Group 5 during fatigue testing. **Conclusions:** Increased demineralization may adversely affect fatigue-based SBS and increase the risk of hard tissue damage. Under plaque-related demineralization conditions, Victory metal and Clarity Advanced ceramic brackets may demonstrate more favorable fatigue bond behavior; however, further in vitro and in vivo studies are required to confirm these findings.

## 1. Introduction

Fixed orthodontic treatment is widely regarded as the gold standard for the correction of dental crowding, malocclusions, and occlusal discrepancies. Its widespread adoption followed the introduction of bracket–archwire systems in the first half of the 20th century, with subsequent diversification of materials and appliance designs driven by technological advances [1]. Early orthodontic brackets were primarily metal and attached to teeth using bands, a technique associated with reduced patient comfort and an increased risk of gingival trauma and sub-band decalcification. The introduction of direct bonding to enamel represented a pivotal shift toward adhesive-based systems, substantially improving clinical efficiency and patient comfort [2]. In parallel with increasing aesthetic demands, ceramic and transparent brackets were developed [3], followed by the introduction of self-ligating bracket systems in the early 1970s, which eliminated the need for ligatures and marked a major evolutionary step in fixed orthodontic therapy [4].

In orthodontics, bonding refers to attaching brackets to the tooth surface using adhesive systems and has largely replaced banded techniques due to reduced treatment time and improved patient comfort. Bonding procedures are classified as direct, in which brackets are placed intraorally, and indirect, where brackets are positioned on a working model and transferred to the dentition using custom trays [5]. In direct bonding, meticulous surface preparation and precise bracket placement are critical for achieving adequate bond strength, as technical shortcomings may lead to bracket failure. Clinically, bracket debonding is commonly associated with bonding errors, consumption of hard or sticky foods, excessive occlusal forces, and incompatibility between the bracket base and the tooth surface [6]. In addition, bracket material and design play an important role in bonding performance, with previous studies reporting higher debonding rates in the maxilla and among female patients [7].

The shear bond strength (SBS) of orthodontic brackets varies depending on bracket type, enamel surface condition, adhesive materials, and the bonding protocol employed. Clinically effective bonding has often been described based on static single-load SBS values reported in megapascals (MPa), with suggested thresholds ranging from 6–8 MPa [8]. While metal brackets typically exhibit debonding strengths of approximately 8–11 MPa, higher values have been reported for ceramic brackets [9]. However, static SBS measurements obtained under single-load conditions may not fully reflect the repetitive functional forces experienced intraorally during orthodontic treatment. Given that orthodontic brackets are exposed to repetitive functional forces rather than a single static load, evaluating bond performance under cyclic conditions may provide a more clinically relevant perspective.

The demineralization process initiated by inadequate removal of dental plaque accumulating around orthodontic brackets has been widely investigated in the literature and represents one of the fundamental mechanisms underlying dental caries and periodontal diseases, which are among the most prevalent oral conditions worldwide [10,11,12]. In fixed orthodontic treatment, brackets, bands, and archwires create retentive sites that facilitate microbial plaque accumulation, leading to low pH conditions that promote enamel demineralization when plaque control is insufficient. Bracket material and design further influence plaque retention, with some studies reporting greater accumulation around ceramic brackets due to surface roughness [13], whereas others have demonstrated higher long-term biofilm formation on metal brackets compared with ceramic counterparts [14].

SBS testing plays an important clinical role in orthodontics by enabling the evaluation of bracket behavior under functional loads, and when performed under cyclic loading conditions, it may provide additional insight into the fatigue behavior of the bracket–adhesive–enamel interface [15]. Given that demineralization is a common finding during fixed orthodontic treatment and may compromise the integrity of this interface, assessing its potential impact on SBS is of particular clinical relevance [16]. Although SBS has been extensively investigated under static loading conditions, comparatively limited evidence exists regarding bracket performance under cyclic fatigue in the presence of enamel demineralization. Moreover, most previous studies have predominantly evaluated bonding on intact enamel surfaces, which may not adequately reflect the structural alterations associated with plaque-induced mineral loss. Therefore, integrating a standardized demineralization model with fatigue-based SBS assessment offers a more clinically relevant framework for evaluating bracket–enamel interface behavior under plaque-related conditions and may provide more realistic guidance for material selection in orthodontic practice.

The aim of this in vitro study was to evaluate the effect of demineralization developing around different types of orthodontic brackets on their fatigue-based SBS, assessed as the number of shear strokes to failure under cyclic loading conditions. The first null hypothesis was that there would be no difference in fatigue-based SBS among the orthodontic brackets. The second null hypothesis was that there would be no correlation between demineralization and fatigue-based SBS.

## 2. Materials and Methods

### 2.1. Sample Size Calculation and Ethical Approval

The sample size was calculated using G*Power software (version 3.1.9.4; Heinrich-Heine-Universität Düsseldorf, Düsseldorf, Germany) and was determined based on the study by Ansari et al. [17]. The effect size was set at 0.71962, with a statistical power of 0.95 and a significance level (*α*) of 0.05. The calculations indicated that a total of 45 samples (9 per group) would be sufficient (actual power: 0.9693164). However, to obtain more robust and statistically powerful results, a larger sample size was used in the present study, with a total of 75 samples (15 per group). Ethical approval for the study was obtained from the Zonguldak Bülent Ecevit University Non-Interventional Clinical Research Ethics Committee (approval date: 31 May 2023; decision no: 2023/11-13).

### 2.2. Specimen Preparation

The specimens used in the present study were derived from a previously conducted specialty thesis completed in 2022 under the supervision of Assoc. Prof. Orhan Cicek, in which orthodontic brackets had been bonded and enamel demineralization was induced through a 28-day exposure to a standardized cariogenic suspension environment [18]. In the original protocol, extracted maxillary premolars were randomly allocated to the experimental groups using a simple randomization method to ensure balanced distribution among bracket types. The bracket-tooth assignment was not predetermined prior to allocation, thereby minimizing potential selection bias.

Specimen inclusion criteria comprised the absence of caries, restorations, cracks, fractures, extraction forceps marks, or enamel fluorosis. Extracted teeth were stored in 0.1% thymol solution at room temperature in a dark environment for a maximum of six months prior to experimentation, with the storage solution renewed monthly [12,18].

Following the 28-day demineralization period, fatigue-based SBS testing was immediately performed by the investigator (T.A.) under standardized mechanical conditions.

In the original bonding protocol, enamel surfaces were cleaned with pumice using a slow-speed handpiece and isolated with a 4 × 4 mm^2^ acetate window to standardize the etched enamel area and bracket positioning at the mesiodistal and inciso-gingival center of the clinical crown. The exposed enamel surface was etched with 37% phosphoric acid for 30 s, rinsed for 15 s, and air-dried for 10 s. A thin layer of Transbond XT primer (3M Unitek, Monrovia, CA, USA) was applied prior to bracket placement. Transbond XT adhesive paste was used for all brackets except adhesive-precoated (APC) brackets, which were bonded according to the manufacturer’s instructions. Excess adhesive was carefully removed before light curing to reduce variability in adhesive thickness. Polymerization was performed for 20 s (10 s mesial and 10 s distal) using an Elipar S10 light-curing unit (3M ESPE, St Paul, MN, USA) [12,18].

Blinding during mechanical testing was not feasible due to the visible morphological differences among bracket types. However, all procedures were performed by the same investigator under standardized conditions to minimize operator-related bias.

Demineralization was assessed using a DIAGNOdent pen (KaVo, Biberach, Germany) calibrated according to the manufacturer’s instructions prior to each session. Measurements were obtained from the gingival, proximal, and occlusal enamel surfaces adjacent to the bracket. During measurement, the probe tip was moved across the enamel surface, and the highest fluorescence value detected was recorded for each reading, consistent with the manufacturer’s recommendations. Two consecutive readings were recorded at each site by the same examiner, and the mean value was calculated to obtain a single demineralization score per specimen, thereby reducing measurement variability. Although DIAGNOdent provides indirect fluorescence-based measurements rather than direct mineral quantification, it is widely used in experimental demineralization models. Averaging values across multiple sites per specimen may reduce site-specific variability; however, localized differences in lesion distribution cannot be entirely excluded [12,18,19].

A total of 75 specimens were included in the study and allocated into five groups, with 15 teeth in each group, as follows:

- **Group 1:** Victory metal brackets (3M Unitek, Monrovia, CA, USA);
- **Group 2:** Adhesive precoated (APC) Clarity Advanced ceramic brackets (3M Unitek, Monrovia, CA, USA);
- **Group 3:** Clarity Self-Ligating ceramic brackets (3M Unitek, Monrovia, CA, USA);
- **Group 4:** Gemini metal brackets (3M Unitek, Monrovia, CA, USA);
- **Group 5:** Clarity Advanced ceramic brackets (3M Unitek, Monrovia, CA, USA).

### 2.3. Acrylic Block Preparation and Specimen Storage

**Figure 1:**
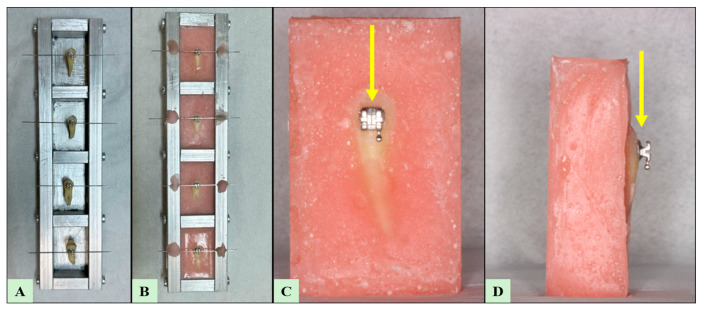
Representative images illustrating specimen preparation, acrylic embedding, and positioning for SBS testing. (**A**) View of tooth specimens during the preparation and stabilization stage prior to acrylic embedding. (**B**) View of the specimens embedded in acrylic blocks following polymerization. (**C**) Frontal view of an individual acrylic-embedded specimen with a bonded orthodontic bracket; the yellow arrow indicates the direction of force application during SBS testing. (**D**) Lateral view of the same specimen demonstrating bracket positioning relative to the acrylic block and the direction of applied shear force (yellow arrow).

**Figure 2:**
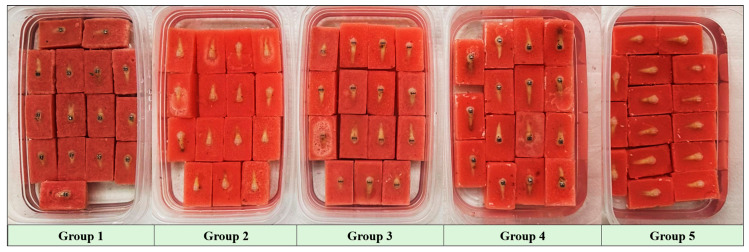
Representative image of acrylic-embedded tooth specimens allocated to five experimental groups according to bracket type prior to fatigue-based SBS testing.

After demineralization, all specimens were removed from the solution, rinsed, and air-dried. Rectangular prism molds measuring 40 × 25 × 15 mm^3^ were used for acrylic block preparation. Prior to embedding, the molds were coated with petroleum jelly to facilitate easy removal of the blocks. The teeth were embedded in self-curing cold acrylic resin (Vertex™, Vertex-Dental B.V., Zeist, The Netherlands) with the tooth surfaces left exposed. During acrylic polymerization, the specimens were stabilized using a 0.019 × 0.025-square inches stainless steel wire secured to the specimens with elastic ligatures and fixed around a metal support frame. After complete polymerization, the specimens were removed from the molds and numbered. The acrylic blocks prepared for shear stroke testing were then stored in distilled water for 24 h, grouped according to the bonded bracket type, until testing was performed (see Figure 1 and Figure 2).

### 2.4. Fatigue-Based SBS Testing Protocol

The primary outcome of the present study was fatigue-based SBS, defined as the number of shear strokes to failure under a constant 10 N cyclic load. Static single-load force-to-failure measurements expressed in MPa were not included in the present study.

**Figure 3:**
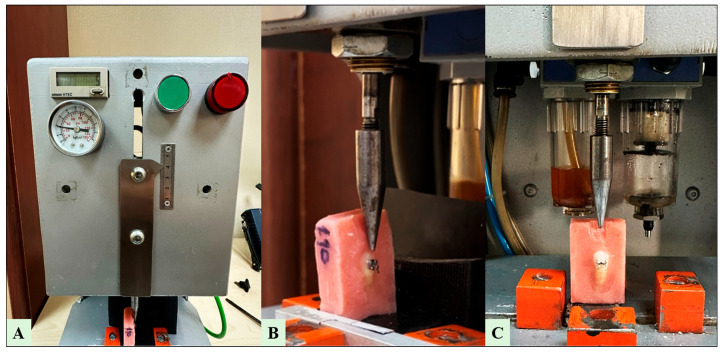
Representative images of the fatigue-based SBS testing setup. (**A**) Front panel of the closed-loop fatigue testing machine. (**B**) Close-up of the acrylic-embedded specimen and square-edged stainless-steel nozzle aligned at 90° to the bracket slot prior to loading. (**C**) Overall configuration during cyclic shear loading, demonstrating standardized fixation and force application parallel to the bracket–enamel interface under a 10 N load.

SBS testing was conducted using a cyclic loading protocol to evaluate the bonding performance of orthodontic brackets under fatigue conditions. The specimens were assessed using a closed-loop controlled, low-cycle fatigue testing machine with a load capacity of 10 N and a crosshead speed of 300 mm/min. During testing, a 90° angle was maintained between the metal nozzle tip and the bracket slot to ensure standardized force application. The acrylic blocks were secured in a custom fixation apparatus, and cyclic loading was applied at a constant force of 10 N until bracket failure occurred. The number of shear strokes to failure was recorded for each specimen (see Figure 3).

**Figure 4:**
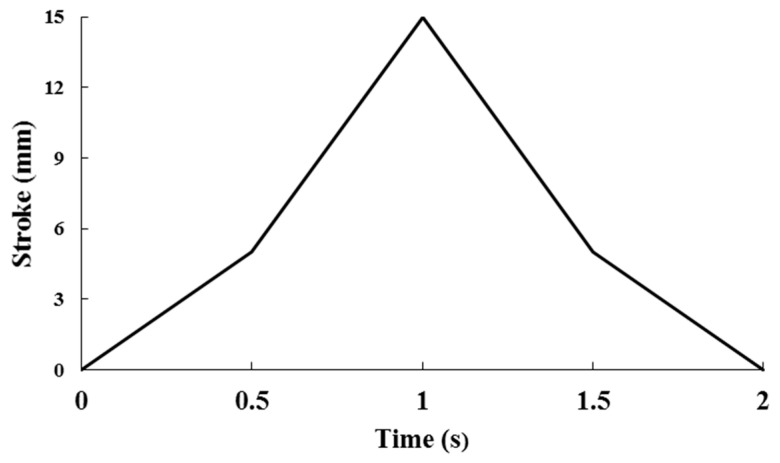
Schematic representation illustrating the definition of one shear stroke under triangular cyclic loading (0–10 N) during fatigue-based SBS testing.

Cyclic fatigue loading was applied at a frequency of 0.5 Hz using a triangular waveform under load-controlled conditions. The load ratio (R) was set to 0 (minimum load = 0 N; maximum load = 10 N). All tests were conducted under dry laboratory conditions at room temperature (23 ± 1 °C). The load was applied in a shear direction parallel to the bracket–enamel interface through a square-edged stainless-steel nozzle positioned at the bracket–adhesive interface. One ‘stroke’ was defined as a complete loading–unloading cycle (0–10–0 N), corresponding to one full triangular waveform cycle, as illustrated in Figure 4.

### 2.5. Hard Tissue Damage Assessment

Following fatigue-based SBS testing, all specimens were examined visually for evidence of hard tissue damage. Evaluation was performed by the same investigator under standardized illumination conditions. Enamel damage was defined as visible surface cracks, loss of enamel structure, or surface discontinuities at the bracket–enamel interface. Dentin involvement was recorded when underlying dentin was visibly exposed following bracket failure.

No magnification devices or microscopic analyses were employed; therefore, assessment was limited to macroscopic evaluation. Hard tissue damage was recorded as present or absent for each specimen. The occurrence of damage was considered to be associated with the fatigue-based shearing process, as specimens were handled using standardized procedures before and after mechanical testing.

### 2.6. Statistical Analysis

Statistical analyses were performed using IBM SPSS Statistics software (version 28.0; IBM Corp., New York, NY, USA). Descriptive statistics were presented as mean, standard deviation, median, minimum, maximum, frequency, and percentage. The normality of the data distribution was assessed using the Shapiro–Wilk test. As the data were not normally distributed, nonparametric statistical methods were applied. Intergroup comparisons were performed using the Kruskal–Wallis test. When statistically significant differences were detected, pairwise comparisons were conducted using the Mann–Whitney U test with Holm–Bonferroni correction to control for type I error due to multiple testing. T0–T1 comparisons within groups were analyzed using the Wilcoxon signed-rank test. The association between enamel demineralization values and fatigue-based SBS (expressed as the number of shear strokes to failure) was evaluated using Spearman’s rank-order correlation coefficient (ρ). Effect sizes for overall intergroup comparisons were calculated using epsilon squared (ε^2^) derived from Kruskal–Wallis test statistics. The level of statistical significance was set at *p* < 0.05.

## 3. Results

At T0, mean demineralization values were comparable among all groups, ranging from 2.18 ± 0.29 in Group 1 to 2.55 ± 0.39 in Group 2, with no statistically significant intergroup differences observed (*p* = 0.145; ε^2^ = 0.04).

Following the demineralization period, a statistically significant increase in demineralization was detected at T1 compared with T0 across all groups (*p* < 0.05). At T1, the lowest mean demineralization value was recorded in Group 1 (Victory metal brackets; 9.48 ± 1.08), followed by Group 2 (APC Clarity Advanced ceramic brackets; 10.91 ± 0.90), Group 3 (Clarity Self-Ligating ceramic brackets; 11.07 ± 1.07), Group 4 (Gemini metal brackets; 11.18 ± 0.79), and Group 5 (Clarity Advanced ceramic brackets; 11.38 ± 1.80). Intergroup comparisons at T1 revealed statistically significant differences (*p* = 0.001), with a moderate effect size (ε^2^ = 0.21). The mean demineralization value in Group 1 was significantly lower than those observed in the other groups (*p* < 0.05), whereas no statistically significant differences were found among Groups 2, 3, 4, and 5 (*p* > 0.05).

The mean number of shear strokes to failure (fatigue-based SBS) was 20.26 ± 19.05 in Group 1 (Victory metal brackets), 8.00 ± 16.67 in Group 2 (APC Clarity Advanced ceramic brackets), 24.93 ± 27.63 in Group 3 (Clarity Self-Ligating ceramic brackets), 7.06 ± 11.60 in Group 4 (Gemini metal brackets), and 30.06 ± 35.31 in Group 5 (Clarity Advanced ceramic brackets). Intergroup comparisons demonstrated statistically significant differences (*p* = 0.001), with a moderate effect size (ε^2^ = 0.14). The mean number of shear strokes to failure was significantly higher in Groups 1, 3, and 5 than in Groups 2 and 4 (*p* < 0.05), whereas no significant differences were observed between Group 1 and Groups 3 or 5, nor between Group 2 and Group 4 (*p* > 0.05).

Statistical analyses of T0 and T1 demineralization measurements and shear stroke numbers for all groups are summarized in Table 1.

**Figure 5:**
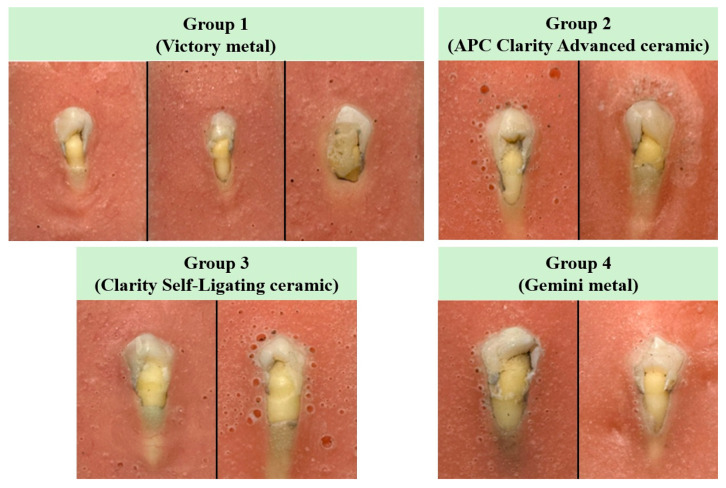
Specimens exhibiting hard tissue damage (enamel and dentin) following fatigue-based SBS testing.

Macroscopic hard tissue damage, defined as visible enamel surface disruption or dentin exposure at the bracket–enamel interface, was observed in 3 specimens in Group 1 (Victory metal), 2 specimens in Group 2 (APC Clarity Advanced ceramic), 2 specimens in Group 3 (Clarity Self-Ligating ceramic), and 2 specimens in Group 4 (Gemini metal). No visible hard tissue damage was detected in Group 5 (Clarity Advanced ceramic) (see Figure 5).

Correlation analysis revealed a statistically significant negative association between demineralization values and the number of shear strokes to failure in Group 2 (ρ = −0.705, *p* = 0.003) and Group 4 (ρ = −0.800, *p* < 0.001). These findings indicate that increasing demineralization was associated with a reduction in the number of shear strokes to failure, corresponding to diminished fatigue-based SBS under cyclic loading. No significant correlations were observed in the remaining groups (*p* > 0.05). Spearman correlation coefficients and corresponding *p*-values for each group are presented in Table 2.

## 4. Discussion

Successful bonding of orthodontic brackets to the enamel surface and the maintenance of adequate bond strength throughout treatment are critical for the continuity and clinical success of fixed orthodontic therapy. Bracket–tooth interface failures represent an inevitable challenge in clinical orthodontics and may compromise treatment efficiency by increasing chair time, prolonging treatment duration, and generating additional costs [20]. Although an ideal bracket failure rate of approximately 6% has been suggested, the literature reports a wide range of failure rates ranging from 0.6% to 28.3% [21]. Moreover, rebonding procedures have been shown to extend overall treatment time by an average of 0.3–0.6 months, while each six-month delay may reduce patient compliance by approximately 23% [22]. In this context, the fatigue-based SBS behavior of orthodontic brackets under functional loading conditions has gained increasing attention in recent years and is considered a clinically relevant parameter for assessing bond performance and predicting long-term clinical behavior [23,24].

The SBS of orthodontic brackets is influenced by multiple factors, including the anatomical location of the tooth, the chemical properties of the adhesive systems used, the condition of the enamel surface, bracket type and base design, surface characteristics, and the bonding protocol applied [20,25]. Any unfavorable condition affecting these factors may compromise bond strength and lead to clinical failures. Although the enamel surfaces to which fixed orthodontic appliances are bonded generally exhibit a low caries prevalence under normal conditions, the presence of orthodontic bands and brackets has been shown to increase plaque retention on otherwise smooth enamel surfaces, thereby predisposing them to demineralization [26]. Accordingly, Almosa et al. [27] reported higher levels of enamel demineralization in bracketed groups compared with non-bracketed controls, while Nee et al. [28] observed significant increases in demineralization on enamel surfaces adjacent to orthodontic brackets after a 12-month follow-up period. Consistent with these findings, the present study demonstrated statistically significant increases in demineralization across all groups at T1 compared with T0 following exposure to a cariogenic environment (*p* < 0.05).

The literature indicates that the material properties of orthodontic brackets may influence the development of enamel demineralization around the bracket. Zawawi and Almosa [29] demonstrated that metal brackets induce deeper enamel demineralization than ceramic brackets under cariogenic conditions. In addition, Bhushan et al. [30] reported that enamel demineralization around orthodontic brackets can be reduced by the use of fluoride-releasing bonding agents, highlighting the multifactorial nature of the demineralization process. In a separate in vitro study, Almosa et al. [27] reported higher levels of enamel demineralization associated with ceramic brackets compared with metal brackets. In the present study, the lowest increase in demineralization at T1 was observed in the Victory metal bracket group, whereas no significant differences were detected among the ceramic bracket groups. Collectively, these findings suggest that enamel demineralization may be influenced not only by bracket material but also by bracket design and bonding-related characteristics.

In vitro testing methods used to evaluate the SBS of orthodontic brackets are widely regarded as reliable because they allow standardized comparisons under controlled experimental conditions [31,32]. Although in vivo studies more closely reflect clinical reality, the presence of numerous uncontrollable variables may complicate data interpretation and limit reproducibility [8,33]. Therefore, the present study employed an in vitro design to obtain more controlled, consistent, and comparable outcomes.

The influence of adhesive agents is among the most extensively investigated factors in orthodontic SBS testing. Transbond XT™ has been widely adopted as a reference adhesive in the literature due to its consistently high bond strength and well-established clinical reliability, with studies by Rai et al. [34] and Shams et al. [35] reporting superior static SBS values compared with alternative materials. Accordingly, Transbond XT™ was used as the standard adhesive in the present study to limit adhesive-related variability and allow a more precise evaluation of bracket-related effects. SBS testing is typically performed by applying a controlled shear force until debonding occurs, using either universal or fatigue testing devices, with reported crosshead speeds ranging from 0.5 to 300 mm/min [36,37,38]. In the present study, a fatigue testing machine operating under load-controlled conditions (maximum 10 N) was employed, enabling cyclic loading conditions that more closely simulate masticatory forces, thereby enhancing the clinical relevance of the experimental protocol. The fatigue protocol was designed to simulate subcritical functional masticatory loading while ensuring reproducibility and comparability with existing in vitro fatigue studies. A 10 N load applied at R = 0 under a triangular waveform (0.5 Hz) generated repetitive shear stress without compressive preload, allowing assessment of bond durability under cyclic conditions rather than immediate debonding force. Accordingly, ‘strokes to failure’ should be interpreted as a measure of fatigue strength under cyclic shear loading rather than as a direct equivalent of static SBS values.

Studies evaluating the SBS of metal orthodontic brackets have highlighted the critical role of bracket base design in bonding performance. Chaudhary et al. [39] reported no significant differences in SBS among Victory metal brackets and various self-ligating metal bracket systems, whereas Corahua-Raymi et al. [40] demonstrated lower bond strength values for brackets with conventional mesh-based designs. In the present study, although both metal bracket groups exhibited mesh-type bases, Victory metal brackets demonstrated a greater number of shear strokes to failure than Gemini metal brackets. This finding may be attributed to differences in base microstructure and manufacturing characteristics that enhance adhesive retention, as well as to the lower degree of demineralization observed in the Victory bracket group, which may have contributed to a more favorable bracket–adhesive–enamel interface behavior under cyclic loading conditions [41].

It is well established that bracket type and base design are key determinants of SBS in ceramic and metal orthodontic brackets. Cicek et al. [36] reported no statistically significant difference in SBS between Clarity SL self-ligating ceramic brackets and Clarity Advanced ceramic brackets, whereas Kavya et al. [42] demonstrated that Clarity Advanced ceramic brackets exhibited significantly higher SBS than Ormco, Koden, and Metro ceramic brackets. Consistent with these findings, a 2021 study reported higher SBS values for Clarity ceramic brackets compared with Gemini metal brackets [43], while Nawrocka et al. [44] found that Clarity Advanced ceramic brackets showed the highest SBS and Victory metal brackets the lowest when bonded using the indirect technique. Similarly, Ansari et al. [17] identified Clarity Advanced ceramic brackets as having the highest mean SBS, followed by APC Clarity Advanced and Gemini metal brackets. In agreement with these findings, the present study demonstrated significantly greater fatigue-based SBS values for Clarity Advanced ceramic brackets than for Gemini metal brackets, which may be attributed to enhanced mechanical retention and more favorable stress distribution associated with their microretentive base architecture under cyclic loading conditions [45]. Our findings demonstrate that the fatigue-based SBS of orthodontic brackets can vary significantly depending on bracket type; accordingly, the first null hypothesis was rejected.

Hard tissue damage to the enamel surface following debonding has been reported more frequently with ceramic brackets than with metal brackets and is commonly associated with the higher bond strength of ceramic systems [46,47,48,49,50]. The literature further indicates that ceramic brackets may induce a greater incidence of enamel cracks and surface damage compared with metal brackets [46,49]. In the present study, hard tissue damage involving both enamel and dentin was observed in specimens from both bracket types; however, no hard tissue damage was detected in the Clarity Advanced ceramic bracket group. This finding may be explained by the microcrystalline base design of Clarity Advanced brackets, which may allow forces to be transmitted to the enamel surface in a more controlled manner [17]. These observations suggest that higher fatigue strength does not necessarily translate into increased enamel damage and may indicate a role of base design in modulating stress distribution.

Although studies investigating the relationship between demineralization and SBS are limited, available evidence suggests that demineralization adversely affects SBS [51,52]. In the present study, higher shear stroke values were obtained in the Victory metal bracket group, which exhibited lower demineralization levels [53,54]. While ceramic bracket groups generally demonstrated higher fatigue-based SBS values in the present study, consistent with trends reported for static SBS in previous studies, the relatively high fatigue-based SBS observed for Victory metal brackets may be attributable, at least in part, to the lower degree of demineralization detected in this group [55]. Overall, a significant negative correlation between demineralization and fatigue-based SBS was demonstrated, leading to the rejection of the second null hypothesis.

### 4.1. Limitations of the Study

The limitations of this study include its in vitro design, which cannot fully replicate intraoral conditions such as temperature fluctuations, salivary flow, and complex occlusal forces. The exclusive use of a single adhesive agent and bonding protocol may limit the generalizability of the findings to other clinical materials or techniques. Furthermore, the absence of a non-demineralized control group precludes direct quantification of the decrease in fatigue-based SBS attributable solely to demineralization; therefore, the results should be interpreted as comparative outcomes among bracket systems under standardized demineralized conditions rather than as absolute measures of demineralization-induced bond loss. Additionally, although DIAGNOdent measurements provide a non-invasive quantitative assessment of enamel demineralization, the use of averaged site-specific values may obscure localized variations adjacent to bracket margins. The lack of Adhesive Remnant Index (ARI) analysis, together with reliance on macroscopic visual inspection without microscopic verification, limits the precision of failure mode characterization and hard tissue damage assessment.

### 4.2. Recommendations for Future Research

Future studies should incorporate non-demineralized control groups to allow direct quantification of the impact of enamel mineral loss on fatigue-based SBS. The use of multiple adhesive agents and bracket designs would further clarify the interaction between material properties and demineralized enamel under cyclic loading conditions. In addition, microscopic or micro-CT–based analyses combined with ARI scoring would provide more detailed insight into enamel integrity and shearing patterns. Finally, well-designed in vitro and in vivo studies conducted under clinically realistic oral conditions are warranted to validate and expand upon the findings of the present study, particularly with respect to fatigue-based SBS.

## 5. Conclusions

The present findings demonstrate that Victory metal brackets exhibited higher fatigue-based SBS than Gemini metal brackets, while Clarity Self-Ligating and Clarity Advanced ceramic brackets showed superior fatigue-based SBS compared with APC Clarity Advanced ceramic brackets. Overall, Victory metal, Clarity Self-Ligating ceramic, and Clarity Advanced ceramic brackets required a greater number of shear strokes to failure, indicating higher fatigue-based SBS under cyclic loading. A significant negative correlation between enamel demineralization and fatigue-based SBS was identified in the APC Clarity Advanced ceramic and Gemini metal groups, suggesting that increased mineral loss may compromise bracket performance under repetitive functional forces. Notably, no visible enamel or dentin damage was observed in the Clarity Advanced ceramic bracket group during fatigue testing. Under plaque-related demineralization conditions associated with fixed orthodontic treatment, Victory metal and Clarity Advanced ceramic brackets may offer more favorable fatigue bond behavior; however, further in vitro and in vivo studies are required to confirm these findings.

## Figures and Tables

**Table 1:** Comparison of T0 and T1 demineralization values and shear stroke numbers among different orthodontic brackets.

Variables		Group 1 ^a^	Group 2 ^b^	Group 3 ^c^	Group 4 ^d^	Group 5 ^e^		
		Mean ± SD (median)	Mean ± SD (median)	Mean ± SD (median)	Mean ± SD (median)	Mean ± SD (median)	*p*	Effect size (ε^2^)
**Demineralization**	T0	2.18 ± 0.29 (2.00)	2.55 ± 0.39 (2.75)	2.43 ± 0.42 (2.50)	2.36 ± 0.48 (2.25)	2.46 ± 0.33 (2.50)	0.145 ^K^	0.04
	T1	9.48 ± 1.08 (9.00) ^b–e^	10.91 ± 0.9 (10.75)	11.07 ± 1.07 (10.75)	11.18 ± 0.79 (11.25)	11.38 ± 1.8 (10.75)	0.001 * ^K^	0.21
	Intra-group *p*	0.001 * ^W^	0.001 * ^W^	0.001 * ^W^	0.001 * ^W^	0.001 * ^W^		
**Shear** **strokes**		**Mean ± SD**	**Mean ± SD**	**Mean ± SD**	**Mean ± SD**	**Mean ± SD**	***p***	**Effect size (ε^2^)**
		20.26 ± 19.05 ^b,d^	8.00 ± 16.67 ^c,e^	24.93 ± 27.63 ^d^	7.06 ± 11.60 ^e^	30.06 ± 35.31	0.001 * ^K^	0.14

SD: standard deviation. *p*: significance value, *: *p* < 0.05. T0: before demineralization. T1: after demineralization. ^K^: Kruskal–Wallis test (pairwise comparisons performed using the Mann–Whitney U test). ^W^: Wilcoxon signed-rank test. ^a^: significantly different from the Victory metal bracket group (*p* < 0.05). ^b^: significantly different from the Gemini metal bracket group (*p* < 0.05). ^c^: significantly different from the Clarity Self-ligating bracket group (*p* < 0.05). ^d^: significantly different from the APC Clarity Advanced bracket group (*p* < 0.05). ^e^: significantly different from the Clarity Advanced bracket group (*p* < 0.05).

**Table 2:** Spearman’s rank-order correlation between enamel demineralization and fatigue-based SBS (number of shear strokes to failure).

Groups	ρ	*p*
Group 1	−0.389	0.151
Group 2	−0.705	0.003 *
Group 3	−0.139	0.622
Group 4	−0.800	<0.001 *
Group 5	−0.168	0.550

ρ: Spearman’s rank-order correlation coefficient. *p*: significance value; *: *p* < 0.05.

## Data Availability

The data that support the findings of this study are available from the corresponding author upon reasonable request.

## Abbreviations

The following abbreviations are used in this manuscript:

SBS	Shear Bond Strength
N	Newton
mm/min	Millimeter per minute
APC	Adhesive Pre-Coated
T0	Before demineralization (Baseline)
T1	After demineralization
SD	Standard Deviation
SPSS	Statistical Package for the Social Sciences
ρ (rho)	Spearman’s correlation coefficient
K	Kruskal–Wallis test
W	Wilcoxon signed-rank test
SL	Self-Ligating
MPa	Megapascal
R	Load ratio
